# mir-193 targets ALDH2 and contributes to toxic aldehyde accumulation and tyrosine hydroxylase dysfunction in cerebral ischemia/reperfusion injury

**DOI:** 10.18632/oncotarget.21129

**Published:** 2017-09-21

**Authors:** Li Mao, Mei-Ling Zuo, Guo-Huang Hu, Xiao-Ming Duan, Zhong-Bao Yang

**Affiliations:** ^1^ ChangSha Health Vocational College, Changsha 410100, China; ^2^ The Affiliated ChangSha Hospital of HuNan Normal University, Changsha 410006, China

**Keywords:** cerebral ischemia/reperfusion injury, miR-193, ALDH2, tyrosine hydroxylase (TH)

## Abstract

MicroRNAs (miRNAs, miR) play a fundamental role in cerebral ischemia/reperfusion (I/R) injury. However, the role of miRNAs in toxic aldehyde and tyrosine accumulation is not fully elucidated. We constructed a cerebral I/R rat model and found that overexpression of miR-193 was associated with the accumulation of 4-Hydroxynonenal (4-HNE), Malondialdehyde (MDA), and tyrosine, and with the decrease of aldehyde dehydrogenase (ALDH2), tyrosine hydroxylase (TH), and dopamine. To unveil the molecular mechanism of the miR-193-mediated phenotype in I/R injury as described above, we performed bioinformatic analysis and found that ALDH2 was a potential target of miR-193. Through *in vitro* experiments (such as miR-193 mimic/inhibitor transfection, luciferase reporter gene plasmid transfection, and 4-HNE exposure) and *in vivo* infusion of miR-193 agomir, we demonstrated that miR-193 directly suppressed the expression of ALDH2 and led to toxic aldehyde accumulation, resulting in dysfunction of tyrosine hydroxylase. The present study suggests that the overexpression of miR-193 in a rat model exacerbated brain injury due to the following sequential process: targeted suppression of ALDH2, aldehyde accumulation, and tyrosine hydroxylase dysfunction, leading to tyrosine accumulation and insufficiency of dopamine synthesis.

## INTRODUCTION

Ischemic stroke is the main cause of death in China, and is associated with a heavy economic and mental burden on patients and their families [1]. Cerebral ischemia triggers a cascade of pathological events that ultimately leads to irreversible neuronal injury in insulted brain tissue [2]. However, the mechanism for brain damage is not yet clear. The development and progression of ischemia/reperfusion (I/R) injury involves the process of oxidative stress, and previous studies have reported that miRNAs play an important role in this process [3, 4]. But, few studies have investigated the relationship between miRNAs and the accumulation of toxic aldehydes and tyrosine. Therefore, further studies on the etiology and pathogenesis of I/R injury are essential.

Emerging evidence indicates that miRNAs act as key modulators of target gene expression, and some, such as miR-21, miR-126, miR-33, miR-125, and miR-222, have been shown to be involved in the pathogenesis of stroke [5, 6]. Several authors have reported altered miRNA expression in rat brains subjected to middle cerebral artery occlusion (MCAO) and 24 hours of reperfusion [5]. MiR-497 was reported to exert its pro-apoptotic effect by suppressing Bcl-2; knockdown of miR-497 attenuated brain infarction in mice [5]. In a previous study, we found that miR-107 significantly increased in MCAO rats and was associated with excitotoxicity [7]. These studies suggest that miRNAs play important roles in cerebral I/R injury. In the present study, we show that miR-193 is significantly increased in rats subjected to 2 hours ischemia and 24 hours reperfusion. Although many authors have reported that miR-193 is involved in the pathological processes of tumors [8, 9], scant information exists on its role in ischemic stroke.

Aldehyde dehydrogenase (ALDH2) is the main enzyme responsible for the oxidization of aldehydic substrates such as 4-HNE, acrolein, and short chain, aromatic, or polycyclic carbons [10]. Previous animal stroke studies have reported that decreased ALDH2 was correlated with higher levels of 4-HNE and MDA [11], and found that ALDH2 protected against stroke by clearing 4-HNE [12]. Aldehydes are very biologically active, having the ability to interact with phospholipids, enzymes, membrane receptors, etc [13]. They play an important role in the process of cerebral I/R oxidative injury [14, 15].

Several studies have previously reported that excess toxic aldehydes influence the function of tyrosine hydroxylase (TH), which is the key rate-limiting enzyme in tyrosine metabolism [11, 12, 29]. Other studies have reported that tyrosine metabolic abnormalities affect the levels of catecholamines and exacerbate brain injury [16–18]. Based on these studies, we hypothesized that accumulation of toxic aldehydes, like 4-HNE, disrupts the function of tyrosine hydroxylase (TH), resulting in abnormal dopamine synthesis and dopamine insufficiency. However, until now, the detailed mechanism for the accumulation of aldehydes and the dysfunction of tyrosine hydroxylase has not been elucidated.

In the current study, we investigated whether overexpression of miR-193 results in the accumulation of toxic aldehydes and tyrosine. We hypothesized that the mechanism of this accumulation involves direct action of miR-193 on ALDH2. To our knowledge, this study is the first to explore the role of miR-193 in cerebral I/R injury and may provide novel targets for the treatment of ischemic stroke.

## RESULTS

### Cerebral ischemia/reperfusion induced brain tissue injury

As shown in Supplementary Figure 1A, the average value of the neurological score was almost 3 points in rats subjected to 2 hours ischemia and 24 hours reperfusion, compared to 0 points in those rats which underwent the sham procedure. The ischemic hemispheres of the I/R group rats were white on TTC staining, indicating that essential metabolic enzymes were inactivated. The infarction volume of the I/R group was up to 0.4 cm^3^ for each ischemic hemisphere, compared to 0 cm^3^ in the sham group (*p* < 0.01, Supplementary Figure 1C), which was expected given the results of the neurological score and TTC staining. H&E staining indicated that brain tissues of the I/R group were marked by edema and cell shrinkage, as compared to controls (Supplementary Figure 1D).

### Cerebral ischemia/reperfusion injury induced increased miR-193, decreased ALDH2, and accumulation of toxic aldehydes

In brain tissues subjected to 2 hours ischemia and 24 hours reperfusion, severe oxidative stress occurs. Cells respond by changing patterns of gene expression. We previously found that the expression of many miRNAs and mRNAs change after I/R injury. One of the affected genes is ALDH2, which responsible for toxic aldehyde metabolism [9].

**Figure 1:**
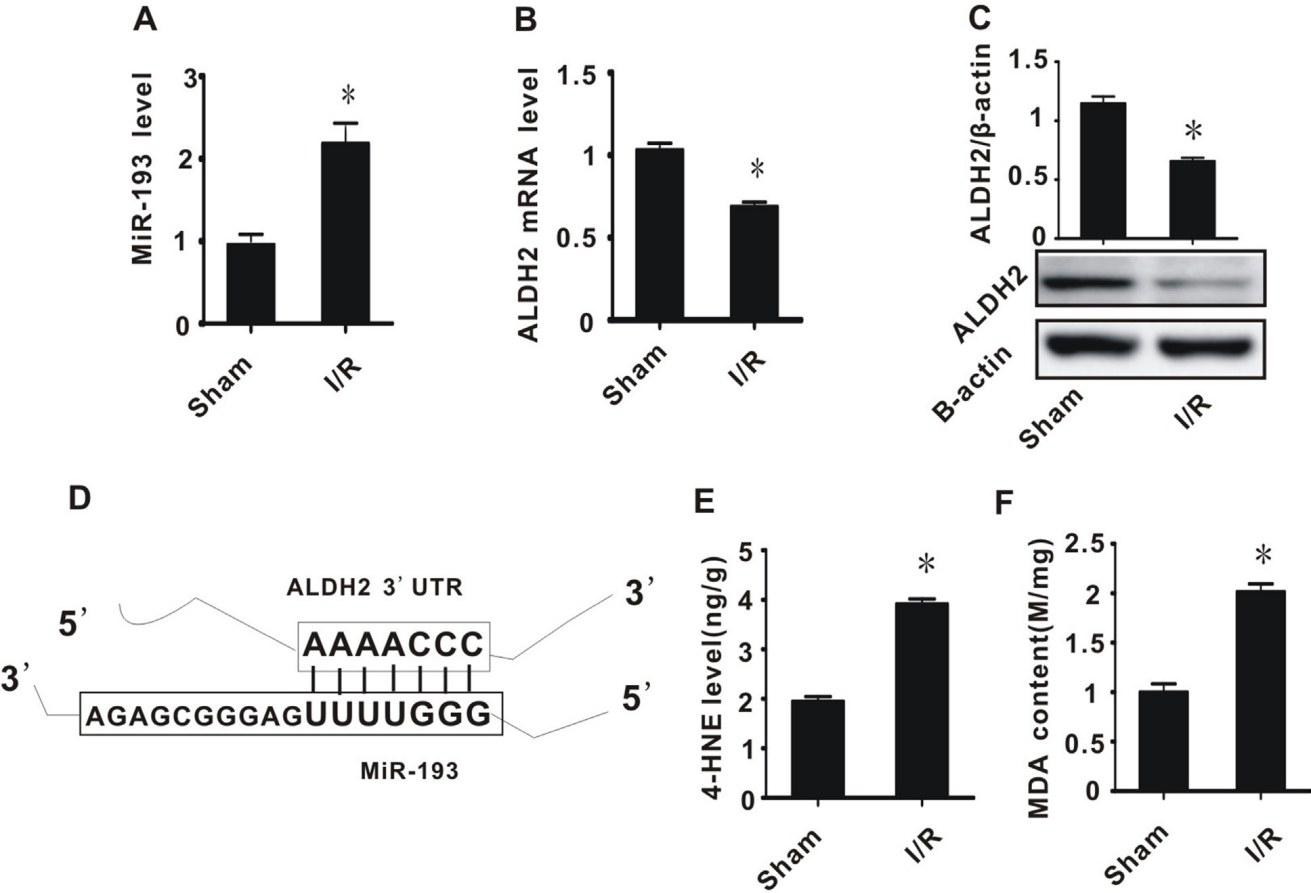
The effect of cerebral ischemia/reperfusion injury on the expression of miR-193, ALDH2, and the accumulation of toxic aldehydes (**A**) miR-193 expression level; (**B**) ALDH2 mRNA level; (**C**) ALDH2 preotein level; (**D**) schematic diagram of interaction between miR-193 and ALDH2; (**E**) 4-HNE content; (**F**) MDA content. All values expressed as means ± S.E.M. I/R: ischemia/reperfusion. ^*^p < 0.05 vs sham.

Bioinformatic analysis revealed that ALDH2 may be a target of miR-193 (Figure 1D). We therefore measured the expression of miR-193 and ALDH2 in a rat model of I/R injury. In the I/R group, the expression of miR-193 significantly increased and ALDH2 (both mRNA and proteins) significantly decreased, compared to the sham procedure group (Figure 1A–1C). Considering that aldehydes are the substrate of ALDH2, we then measured the content of MDA and 4-HNE, which are both classic toxic aldehydes generated during the process of oxidative stress. In the I/R group, both MDA and 4-HNE were significantly increased compared to the sham group (Figure 1E and 1F). Taken together, these data suggest that, after I/R injury in rats, there is an increase in miR-193, a decrease in ALDH2, and an increase in toxic aldehyde accumulation.

### Cerebral ischemia/reperfusion injury contributed to decreased TH and abnormal tyrosine metabolism

**Figure 2:**
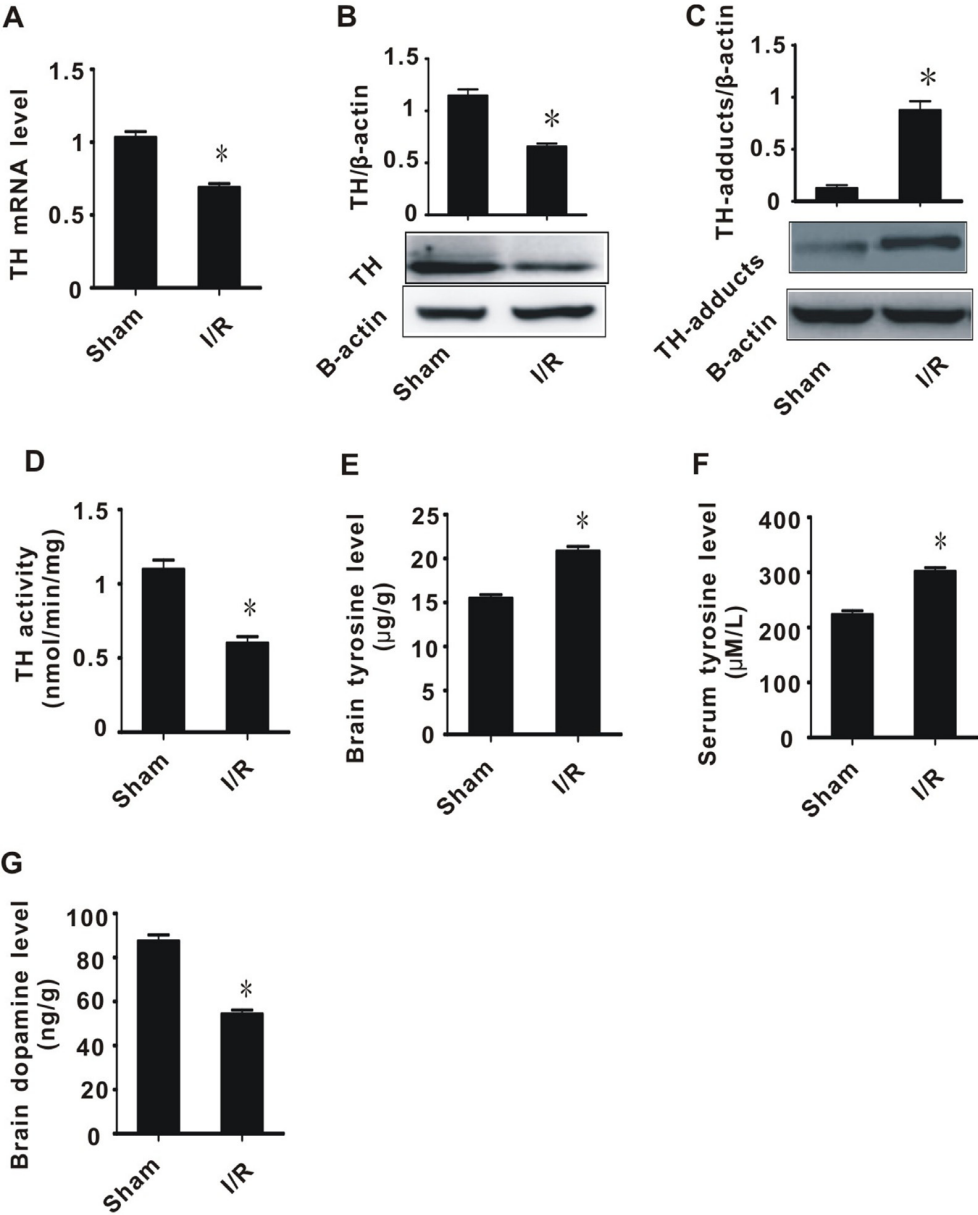
The effect of cerebral ischemia/reperfusion injury on TH and tyrosine metabolism (**A**) TH mRNA level; (**B**) TH protein level; (**C**) TH-adducts level; (**D**) TH enzyme activity; (**E**) tyrosine content in brain tissue; (**F**) tyrosine content in plasma; (**G**) Dopamine content. All values expressed as means ± S.E.M. I/R: ischemia/reperfusion. ^*^p < 0.05 vs sham.

4-HNE is an aldehyde, and as such, can induce the degradation of many biological macromolecules, which can in turn result in tissue damage. This process is generally known as lipid peroxidation, and plays a pivotal role in the development and progression of cerebral I/R injury. To explore whether I/R injury and 4-HNE affects the expression of TH, we performed real-time PCR and western blot assays. In I/R rats, the expression and activity of TH significantly decreased, while 4-HNE/TH adducts significantly increased, compared with the sham group (Figure 2A–2D).

Since TH is a key enzyme for tyrosine metabolism, we hypothesized that, after I/R injury, abnormal TH contributes to tyrosine accumulation and insufficiency of dopamine synthesis. We therefore measured the tyrosine and dopamine levels after I/R injury. The I/R group had significantly higher tyrosine levels in both brain tissue and plasma, and lower dopamine levels in brain tissues, compared with the sham group (Figure 2E–2G). These data suggest that cerebral I/R injury leads to a decrease in TH activity, an increase in tyrosine levels, and a decrease in dopamine levels.

### Exogenous miR-193 mimics suppressed ALDH2 expression and increased aldehyde levels in PC-12 cells

**Figure 3:**
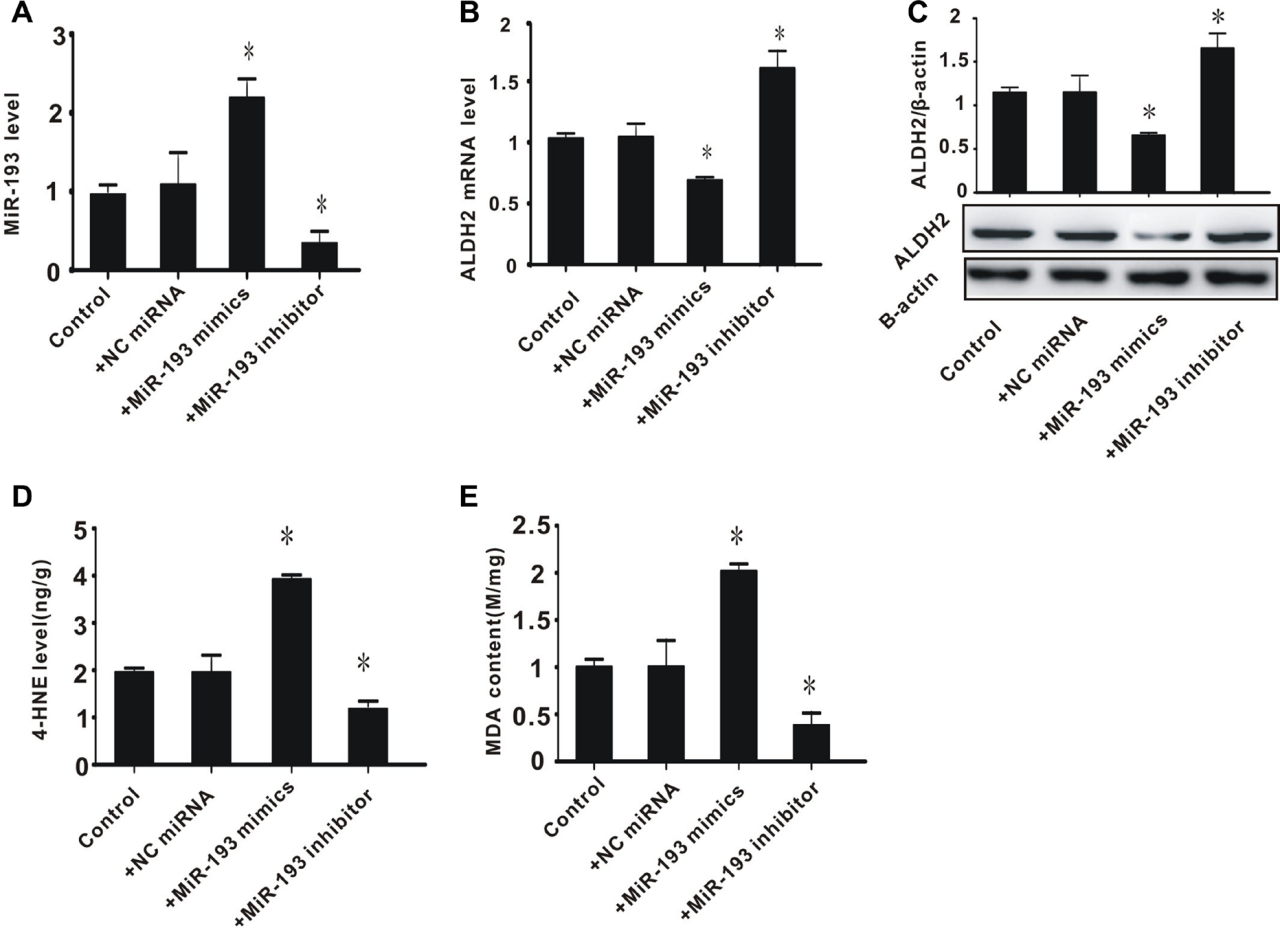
The effect of exogenous miR-193 mimics on ALDH2 expression and aldehyde levels in PC-12 cells (**A**) miR-193 expression level; (**B**) ALDH2 mRNA level; (**C**) ALDH2 protein level; (**D**) 4-HNE content; (**E**) MDA content. All values expressed as means ± S.E.M. +NC control miRNA: negative control miRNA. ^*^p < 0.05 vs control.

To determine whether high expression of miR-193 is associated with excessive aldehyde levels, PC-12 cells were treated with miR-193 mimics or an miR-193 inhibitor. As shown in Figure 3A, transfection with miR-193 mimics significantly increased miR-193 expression, while the miR-193 inhibitor significantly decreased miR-193 expression, compared to controls. We then measured the mRNA and protein expression of ALDH2. Consistent with our results from the rat model (Figure 1), miR-193 mimics significantly suppressed ALDH2 expression, while the miR-193 inhibitor significantly increased the expression of ALDH2 (Figure 3B and 3C).

With regard to toxic aldehyde levels, we found that miR-193 overexpression significantly increased the levels of toxic aldehydes 4-HNE and MDA, while the miR-193 inhibitor significantly decreased these levels, compared to controls (Figure 3D and 3E). These data indicate that, consistent with our observations in rats, overexpression of miR-193 is associated with decreased expression of ALDH2 and increased accumulation of toxic aldehydes, while inhibition of miR-193 leads to increased expression of ALDH2 and a decrease in toxic aldehyde accumulation.

### Exogenous miR-193 mimics decreased expression of ALDH2 in a luciferase reporter gene construct

**Figure 4:**
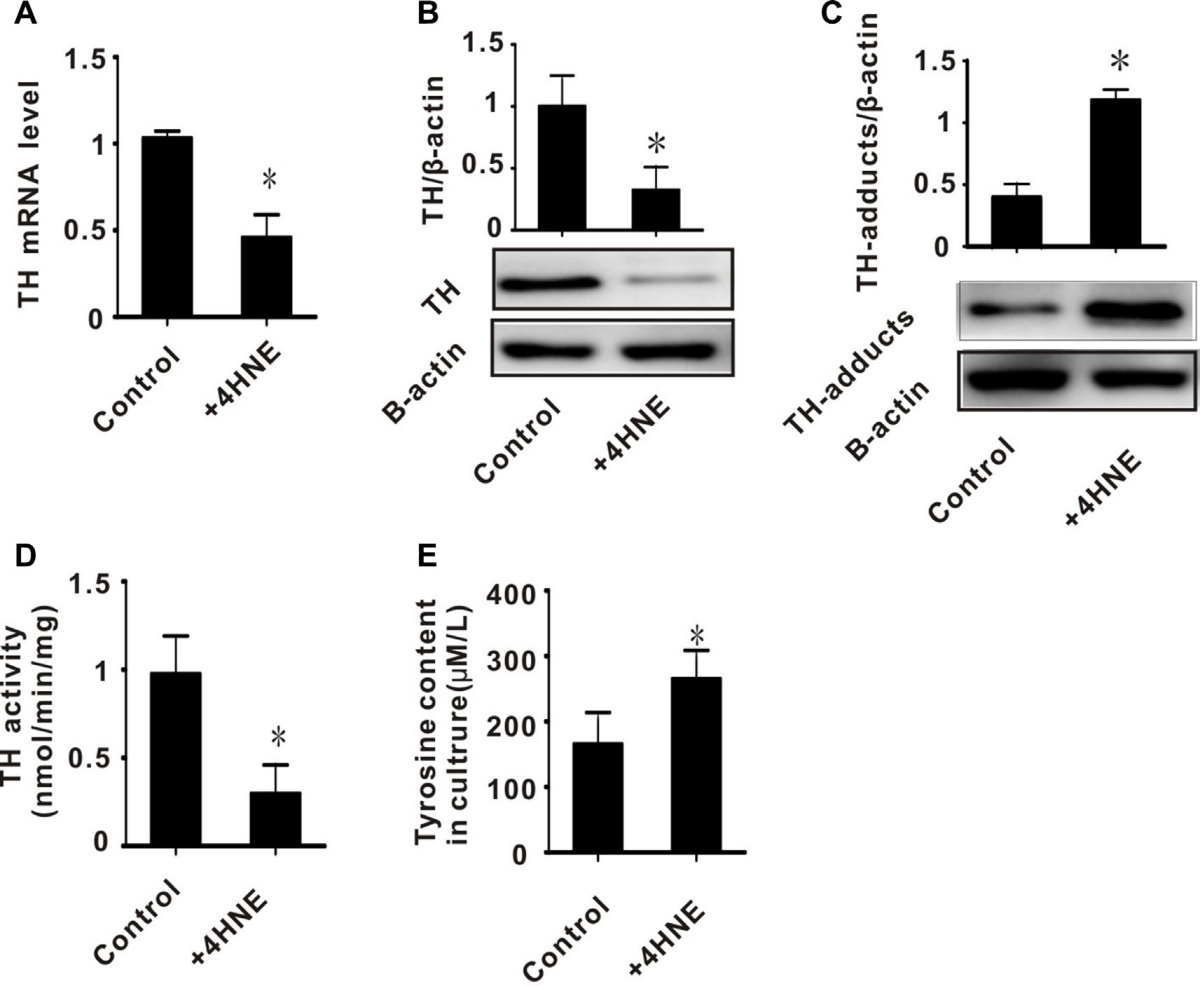
The effect of exogenous miR-193 mimics on relative luciferase activity +NC: negative control plasmid; +ALDH2-WT: plasmid containing wild type of 3′UTR of ALDH2; +ALDH2-MU: plasmid containing wild type of 3′UTR of ALDH2. All values expressed as means ± S.E.M. ^*^p < 0.05 vs +NC with +miR-193 mimics, ^#^*p* < 0.05 vs +ALDH2-WT with +miR-193 mimics.

To determine whether miR-193 regulates ALDH2 activity, a reporter gene was constructed as described in the methods section. Plasmids containing the wild type 3′UTR of ALDH2 (ALDH2-WT, with the seed sequence “AAAACCC”), or mutated 3′UTR of ALDH2 (ALDH2-MU, with the seed sequence “AAAGCCC”), were co-transfected with the miR-193 mimics and/or inhibitor into PC-12 cells. As shown in Figure 4, miR-193 mimics significantly decreased the relative luciferase activity in the ALDH2-WT group but not in the ALDH2-MU group. Furthermore, the miR-193 inhibitor reversed the suppression caused by miR-193 mimics. These data indicate that miR-193 regulates the expression of ALDH2.

### Exogenous 4-HNE induced abnormalities of tyrosine hydroxylase in PC-12 cells

**Figure 5:**
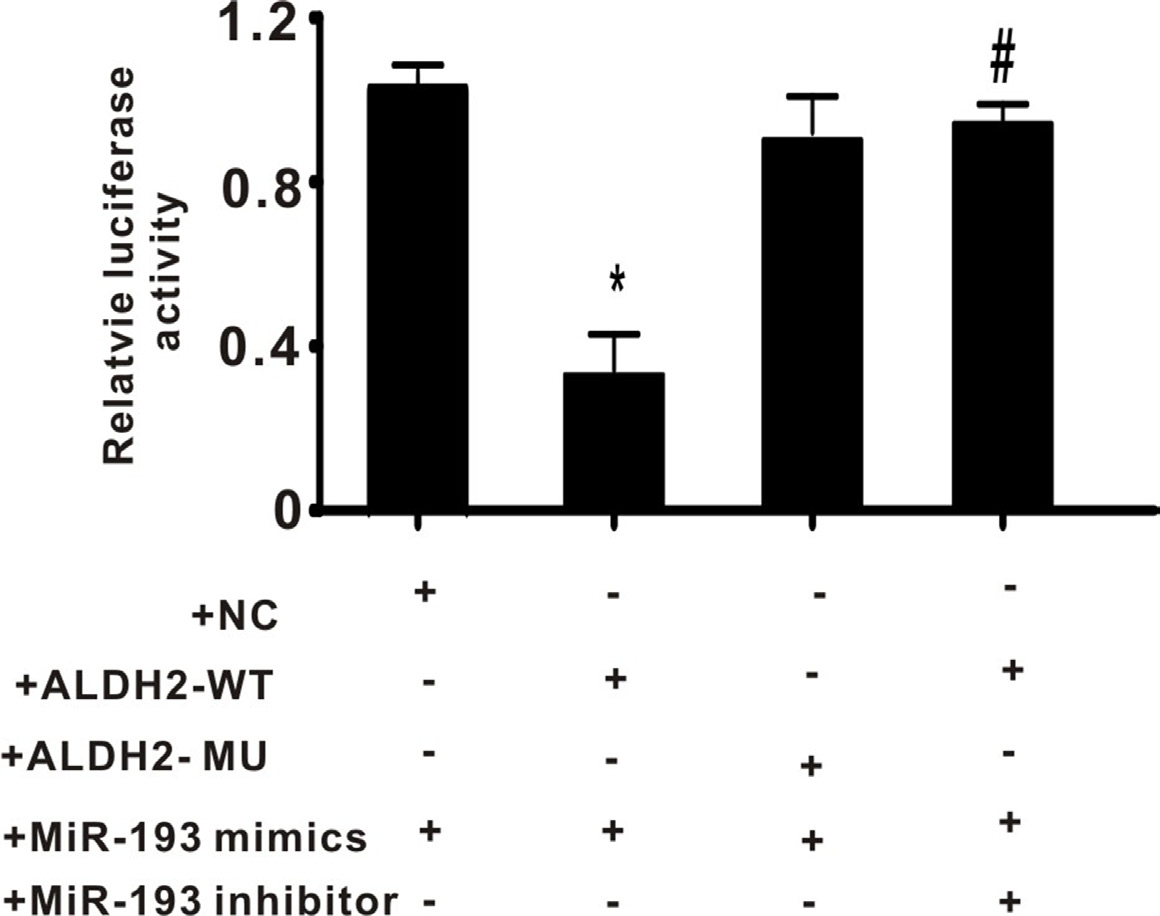
The effect of exogenous 4-HNE on tyrosine hydroxylase in PC-12 cells (**A**) TH mRNA level; (**B**) TH protein level; (**C**) TH-adducts level; (**D**) TH enzyme activity; (**E**) tyrosine content in culture. All values expressed as means ± S.E.M. +4-HNE: 50 μM 4-hydroxynonenal. ^*^p < 0.05 vs control.

In rats, we found that the accumulation of toxic aldehydes was inversely related to the expression and activity of TH (Figures 1D, 1E, 2A, 2B, and 2D). We also found increased 4-HNE/TH adducts in I/R injured brain tissues (Figure 2C). We therefore hypothesized that 4-HNE might lead to decreased expression of TH. To test this, PC-12 cells were treated with 50 umol/L 4-HNE. We then measured the expression and activity of TH, as well as the level of 4-HNE/TH adducts. The expression and activity of TH in PC-12 cells treated with 4-HNE were significantly decreased, while TH/4-HNE-adducts in these cells were significantly increased (Figure 5A–5D). To confirm the effect of TH on tyrosine metabolism, we measured the content of tyrosine in these cultures and found a higher tyrosine content in the 4-HNE treated group (280 μM/L), as shown in Figure 5E. These results suggest that toxic aldehydes such as 4-HNE affect the function of tyrosine hydroxylase through addition reactions and thereby influence tyrosine metabolism.

### MiR-193 agomir injection into rats contributed to inhibition of ALDH2 expression, accumulation of 4-HNE, inhibition of TH expression and activity, accumulation of tyrosine, and decrease of dopamine

**Figure 6:**
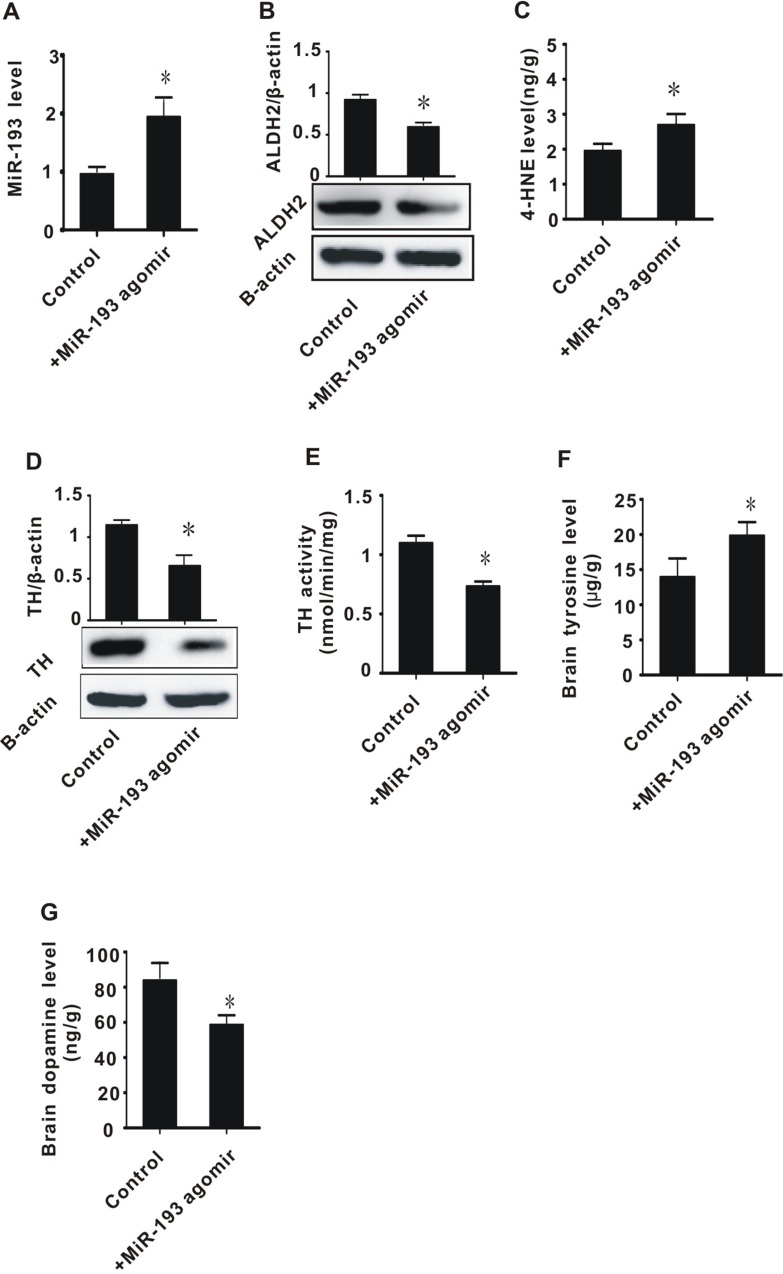
The effect of *in vivo* infusion of miR-193 agomir on brain tissues (**A**) miR-193 expression level; (**B**) ALDH2 protein level; (**C**) 4-HNE content; (**D**) TH protein level; (**E**) TH enzyme activity; (**F**) tyrosine content in brain tissue; (**G**) Dopamine content. +MiR-193 agomir: tail vein infusion with miR-193 agomir (50 nmol/day) for two days. All values were expressed as means ± S.E.M. I/R: ischemia/reperfusion; ^*^p < 0.05 vs sham.

Considering the important role of miR-193 in cerebral ischemia/reperfusion injury, we conducted an *in vivo* experiment to explore the effects of miR-193 agomir on toxic aldehyde accumulation and tyrosine metabolism in rats. To ensure the expected functioning of miR-193 agomir, we measured miR-193 expression before and after injection with miR-193 agomir and found that miR-193 expression significantly increased in rats treated with miR-193 agomir (Figure 6A). We then measured the expression of ALDH2 before and after injection of miR-193 agomir and found a significant decrease in ALDH2 levels after miR-193 injection (Figure 6B). Rats treated with miR-193 agomir had significantly increased levels of 4-HNE, decreased expression and activity of TH, increased tyrosine levels, and decreased dopamine levels (Figure 6C–6G). These data are consistent with the results of the cerebral I/R *in vivo* experiments and *in vitro* cell experiments described above.

## DISCUSSION

In this study, we found that the expression of miR-193 was significantly increased in rats with cerebral ischemia/reperfusion injury. This injury was associated with the accumulation of toxic aldehydes and tyrosine (Figures 1 and 2). We demonstrated that miR-193 directly inhibited the expression of ALDH2, which is partly responsible for clearance of toxic aldehydes. Overexpression of miR-193 *in vitro* and in a rat model led to increased toxic aldehyde accumulation. We found that the accumulation of toxic aldehydes, such as 4-HNE, contributed to the inhibition of tyrosine hydroxylase expression and resulted in tyrosine accumulation. Taken together, these data indicate that cerebral I/R injury is associated with overexpression of miR-193, which, through its action on ALDH2, drives toxic aldehyde and tyrosine accumulation and subsequent dopamine insufficiency (Figure 1 and Supplementary Figure 1).

As an important regulatory factor, miR -193 has a variety of biological functions and is involved in the pathogenesis of tumor, hypertension, stroke, and other diseases [8, 9]. Decreased expression of miR-193 is usually associated with tumorigenesis, such as oral squamous cell carcinoma, acute myeloid leukemia, lung cancer, BRAF-mutated melanoma, etc [8]. Williams *et al.* found that overexpression of mir-193a-3p inhibited malignant pleural mesothelioma (MPM) xenograft tumor growth by directly suppressing myeloid cell leukemia-1 (MCL1) expression, and was associated with increased apoptosis [8]. MiR-193a-3p is also involved in multi-drug resistance or multi-chemoresistance of tumors, via presenilin 1 (PSEN1) gene or lysyl oxidase-like 4 (LOXL4) gene suppression [9]. According to Wu *et al*, deregulation of miR-193b affected cell growth in colon cancer via the TGF-β and SMAD3 signaling pathways [19]. In addition, altered expression of miR-193 is also found in cardiovascular and cerebrovascular diseases. Many studies have described downregulated miR-193 in rat cerebral endothelial cells after stroke [20]. However, in the present study, we found that in rats subjected to 2 hours ischemia and 24 hours reperfusion, miR-193 significantly increased. These results are consistent with the results described by Ky *et al.* [21–23]. We therefore think that miR-193 plays an important but understudied role in cerebral I/R injury. In the present study, we found that ALDH2 is a potential target of miR-193 and demonstrated that miR-193 inhibits ALDH2 by directly targeting its 3′UTR (Figures 3 and 4). These data suggest that miR-193 plays a pivotal role in oxidative stress.

Aldehyde dehydrogenase 2 (ALDH2) is an important mitochondrial enzyme responsible for the detoxification of aldehydes, which mainly depends on ALDH2-mediated oxidation of reactive aldehydes (high toxicity) to carboxylic acids (low toxicity) [24]. Our results echo several previous studies which have shown that ALDH2 is involved in the process of I/R injury or ischemic stroke [10, 12, 14]. Given the important role of ALDH2 in oxidative stress, elucidating the mechanism of its regulation, especially post-transcriptionally, is one key to understanding ischemic stroke. Previous studies have demonstrated that miRNAs control diverse aspects of brain disease, including cerebral I/R injury [25, 26], and that miRNAs are involved in regulation of ALDH2 expression. For example, Li *et al.* found that miR-28 promotes cardiac ischemia by targeting mitochondrial ALDH2 in mouse cardiac myocytes [27], and Fan *et al.* found that miR-34a promotes cardiomyocyte apoptosis through down-regulating ALDH2 [28]. However, the detailed mechanism for this regulation has not yet been fully elucidated. Considering that one mRNA can be regulated by several different miRNAs, we hypothesized that ALDH2 is simultaneously regulated by different miRNAs other than miR-28 and miR-34a. In the present study, we demonstrated that miR-193, which is overexpressed after cerebral I/R injury, directly suppresses ALDH2 (Figures 2–4).

Decreased expression of ALDH2 in I/R injury results in the accumulation of toxic aldehydes. 4-HNE and MDA, which are markers of oxidative stress, are the most commonly generated toxic aldehydes [14, 15]. Several studies have previously reported that excess toxic aldehydes influence the function of tyrosine hydroxylase (TH), which is the key rate-limiting enzyme in tyrosine metabolism [11, 12, 29]. Our study found that, in I/R injured rats or exogenous 4-HNE challenged PC-12 cells, the expression and activity of tyrosine hydroxylase significantly decreased, and was inversely related to 4-HNS and MDA levels. This suggests that lipid peroxidation affects the metabolism of tyrosine (Figures 1, 2, and 5). We also found that the levels of tyrosine in rats with I/R injury significantly increased, while the levels of dopamine significantly decreased (Figure 2). These results suggest that one of the important causes of tyrosine accumulation and catecholamine insufficiency in cerebral I/R injury is toxic aldehyde-induced TH dysfunction.

Tyrosine, the substrate of TH, can be converted to dihydroxy phenylalanine; thus, tyrosine is essential for the synthesis of neuronal catecholamines. Catecholamines are converted to dopamine by dopamine decarboxylase, and are then transported into vesicles in which norepinephrine is generated via beta- hydroxylation [16]. Abnormal tyrosine metabolism therefore directly affects the synthesis and function of catecholamines, and worsens the outcome of ischemic stroke and other neurological diseases [1, 16, 17]. In the present study, we found that the expression and enzymatic activity of TH were significantly reduced in a rat model of ischemic stroke (Figure 2). This indicates that TH plays an important role in the development of ischemic hypoxic brain damage [17]. In fact, many clinical studies have shown that the tyrosine concentration and the ratio of phenylalanine to tyrosine significantly increases in the serum of patients with ischemic stroke [30]. Also, in post-stroke rehabilitation, administration of dopamine can significantly improve motor function [31, 32]. This suggests that neurotransmitter dysfunction is one potential cause of nerve cell damage, and the amount of neurotransmitter dysfunction may therefore affect patient prognosis [17, 35].

In conclusion, we have shown that overexpression of miR-193 in cerebral I/R injury was associated with the accumulation of toxic aldehydes and tyrosine. We explored the relationships between the metabolic abnormalities of aldehydes and tyrosine in cerebral I/R injury, and found that the underlying mechanism involved is the miR-193/ALDH2/4-HNE/TH/dopamine axis. Much work remains to fully elucidate the pathogenesis of cerebral I/R injury. This study may provide novel targets for ischemic stroke therapy and drug development.

## MATERIALS AND METHODS

### Animal experiments

Male Sprague–Dawley (SD) rats weighing 250–300 g were purchased from Hunan SJA Laboratory Animal Co., Ltd. Animals were housed for a week to accommodate to the environment. Before experiments, the rats fasted for 24 h, but had free access to tap water. The study was performed following the Guide for the Care and Use of Laboratory Animals, published by the National Institutes of Health (NIH Publication No. 85–23, revised 1996) and experiments were approved by the HuNan Normal University Veterinary Medicine Animal Care and Use Committee.

The rat model of I/R injury was established according to our lab’s method, which has been described elsewhere [7]; briefly, the middle cerebral artery of the rat was occluded (MCAO) for 2 h with subsequent reperfusion for 24 h. The body temperature of the rat was maintained at ∼37°C throughout the procedure.

The animals were randomly allocated to two groups (*n* = 8 per group): the sham group, which underwent surgical procedures but had no ischemic insult, and the I/R group, which was subjected to 2 h of ischemia followed by 24 h of reperfusion. Animals from the sham group underwent the same procedure as the I/R group except that the occluding filament was inserted only 7 mm above the carotid bifurcation.

At the end of reperfusion, the neurological deficit score was assessed, then the plasma was collected for measurement of tyrosine levels, and finally the brain tissue was saved for infarct volume measurement or other measurements (such as levels of expression of mRNA or protein).

### Assessment of neurological deficit score and measurement of infarct volume

A neurological score was used to evaluate loss of neurobiological function according to the following 5-point rating scale: 0 = no deficit, 1 = failure to extend the left forepaw, 2 = decreased grip strength of left forepaw, 3 = circling to left by pulling the tail, 4 = spontaneous circling [33].

For measurement of infarct volume, the brains were sectioned into 4 coronal sections of 0.2-0.3 cm thickness. The sections were immersed in 2% 2,3,5-tripenyltetrazolium chloride (TTC) for 30 minutes at 37°C, then images were scanned into a computer and measured with imaging analysis software (Image J, NIH, USA). The presence or absence of infarction was determined by examining the TTC stain. The infarct volume (in cm3) for each section was equal to infarct area (in cm2) multiplied by the section thickness (0.2 or 0.3 cm). The total infarct volume for each brain was then calculated by summing up the infarct volume of all sections. To minimize the effect of edema on the accuracy of infarct volume measurement, the final infarct volume was corrected by a factor equal to the ratio of non-ischemic to ischemic hemisphere volumes.

### Cell culture

The PC-12 cell line was purchased from the Committee on Type Culture Collection of the Chinese Academy of Sciences of ShangHai. Cells were cultured in DMEM medium supplemented with 10% FBS and penicillin/streptomycin in a 95% air and 5% CO2 atmosphere. Cells were subcultured and seeded into 6- or 24-hole cell plates for miRNA mimic or inhibitor transfection experiments. Finally, cells were digested in 0.2% trypsogen for mRNA or protein analysis.

### Bio-informatics analysis

The target genes of miRNAs and the target miRNAs of genes were predicted by the online systems TargetScan and miBase. Because ALDH2 is significantly decreased in brain tissue subjected to I/R injury while miR-193 is significantly increased, we focused on these.

### Exogenous 4-HNE challenging

Exogenous 4-HNE was used to investigate the effect of toxic aldehydes on tyrosine hydroxylase. Briefly, PC-12 cells were seeded into 6-well plates and held in 20% O2, 5% CO2, at 37°C in DMEM culture supplemented with 10% fetal bovine serum. When the cells had grown to a confluence of 80%, 50μM 4-HNE was added. After being exposed to 4-HNE for 24 hours, cells were collected for mRNA or protein analysis.

### MiR-193 mimic and inhibitor transfection

MiR-193 mimic or inhibitor transfection experiments were conducted according to the instructions of the manufacturer. Before transfection, a mix including the transfection agent lip2000 and miR-193 mimics or inhibitor were prepared. The mix was added into a 24-well plate to make a final concentration of 100 nmol. Cells were exposed to this mix for 6 hours. Finally, the cells were collected for mRNA or protein analysis.

### Luciferase reporter gene experiment

To further confirm the interaction between miR-193 and ALDH2, a sequence including the seed region sequence was cloned into plasmid GV272 to construct a luciferase reporter gene. Two plasmids were constructed: first, a wild type (ALDH2-WT) with the seed region sequence of “AAAACCC,” and second, a mutant (ALDH2-MU) with the seed region sequence of “AAAGCCC.” To evaluate the interaction of miR-193 and ALDH2, these plasmids, together with miRNA mimics, were co-transfected into PC-12 cells and the relative luciferase activity was detected.

### Intracerebroventricular infusion of miR-193 agomir

To explore whether miR-193 overexpression is one of the factors of cerebral I/R injury, an *in vivo* experiment was conducted. In accordance with the manufacturer’s instructions, miR-193 agomir was dissolved in PBS at a concentration of 50 nmol/ml. Then, 5 nmol of the miR-193 agomir solution was injected into rats’ right lateral ventricle of the brain following the methods described by Fei Zhu et al. for each of two consecutive days. Two days after the final Intracerebroventricular infusion, the rats were anesthetized and killed after which their tissues were analyzed with real-time PCR, western blot and other biochemical analysis such as tyrosine content.

### Measurement of TH mRNA expression

Real-time PCR was used to analyze TH mRNA levels in brain tissue. Total RNA was extracted by using the TRIzol reagent (TaKaRa, Dalian, China) and the concentration and purity of RNA was determined spectrophotometrically. Two hundred ng of RNA from each sample was used for the reverse transcription reaction, which was conducted using a transcription kit (DRR037A; TaKaRa, Dalian, China). Real-time PCR (ABI 7300) was used to quantitatively determine the TH mRNA expression levels using SYBR Premix Ex Taq (TaKaRa Dalian, China). The real-time PCR primers for TH and β-actin are displayed in Table 1. Data analysis was performed with the comparative Ct method using the ABI software. The result was adjusted by the ratio of TH mRNA to β-actin mRNA.

**Table 1:** Primers for real-time PCR

Gene	Forward primer	Reverse primer	Product size (bp)
TH	5′-CGACCTCGAGATCCATTGTGC-3′	5′-ACTATCATTAAGGACCCAGGGC-3′	157
β-actin	5′-CCCATCTATGAGGGTTACGC-3′	5′-TTTAATGTCACGCACGATTTC-3′	150
ALDH2	5′-CACGGAAGTGAAGACGGTCA-3′	5′-CCAGACGCTTTGGTGAAGGG-3′	163

### Determination of TH protein expression

The total protein of each sample was extracted using the cell lysis buffer for Western and IP (Beyotime, China). Forty μg protein was used for western blot analysis according to the following steps: Proteins were run on an SDS-PAGE (10% gel), then transferred to polyvinylidene fluoride (PVDF) membranes. They were incubated with rabbit anti-TH (Santa Cruz, CA, USA) followed by horseradish peroxidase-conjugated secondary antibodies. We determined the signals of bands using enhanced chemiluminescence (ECL kit, Amersham Biosciences, Piscataway, NJ, USA) and the Molecular Imager ChemiDoc XRS System (Bio-Rad, Philadelphia, USA). The densitometric analysis was conducted with Image J 1.43 (National Institutes of Health). To ensure equal loading, blots were incubated with mouse anti-β-actin (Millipore, Billerica, MA, USA).

### Determination of TH enzyme activity

TH enzyme activity was determined using a commercial ELISA kit. According to the manufacturer’s instructions, the Microelisa stripplate provided in this kit had been pre-coated with an antibody specific to TH. Standards or samples were added to the appropriate Microelisa stripplate wells and combined to the specific antibody. Then, a Horseradish Peroxidase (HRP)- conjugated antibody specific for TH was added to each Microelisa stripplate well and incubated. Free components were washed away. The TMB substrate solution was added to each well. Those wells that contained TH and HRP-conjugated TH antibody appeared blue in color, and then turned yellow after the addition of the stop solution. The optical density (OD) was measured spectrophotometrically at a wavelength of 450 nm. The OD value was proportional to the activity of TH, which was calculated by comparing the OD of the samples to a standard curve, and expressed as U/ml.

### Measurement of 4-HNE and MDA contents

4-HNE content was measured using a commercial ELISA kit (R&D, Minneapolis, USA). Briefly, 100 μl of tissue lysates from each sample were added to a 96-well protein binding plate and incubated at 37°C for 2 h. Then, anti-HNE-His primary antibody and HRP-conjugated secondary antibody were successively added to probe for the 4-HNE protein adducts. After adding stop solution, the absorbance of each well was immediately read at 450 nm and the HNE-protein content, expressed as ng/g protein, was determined by comparing the absorbance with a standard curve.

The MDA contents were measured using the thiobarbituric acid (TBA) method with slight modification (Beyotime, Shanghai, China) [12]. Briefly, 0.5 ml of tissue lysates were mixed with 3 ml of 1% phosphoric acid and 1 ml of 0.67% TBA. The mixture was incubated at 95°C for 60 min and subsequently cooled. In order to extract the MDA, 375 µl N-butanol was added and vortexed vigorously for 10 s. After centrifuging, the upper N-butanol layer was transferred to a glass tube. The absorbance of the butanol phase was measured at 532 nm. The MDA content was expressed as mmol/mg protein.

### Measurement of tyrosine and dopamine contents

The tyrosine content was measured using a commercial kit (SIGMA, MAK219) and following the manufacturer’s instructions [34]. Briefly, we made a 2.5 mM tyrosine standard solution and added 0, 2, 6, 12, 18, and 24 μl to a 96-well plate. We then added water to each well to bring the volume to 150 μl, making 0, 5, 15, 35, 45, 60, and 75 nmol/well standards. To another 96-well plate, we added 100 μl of the prepared samples followed by water to bring the volume in each well to 150 μl. We then added 50 μl of the reaction mixture to the standard and sample wells. We incubated and agitated the wells for 60 minutes, in the dark, at a regular temperature. The absorbance of each well at 492 nm was immediately read and the tyrosine content was expressed as μg/g protein.

The dopamine content was measured using a commercially available ELISA kit (Ractopamine, RAC). Briefly, 50 μl of standard or sample was added to a 96-well protein binding plate and incubated at 25°C for 30 min in the dark. Anti-Ractopamine primary antibody and HRP-conjugated secondary antibody were successively added to make a Ractopamine-enzyme complex. TMB substrate was added and, after adding stop solution, the absorbance of each well at 450 nm was immediately read. The dopamine content was expressed as ng/g protein.

### Statistical analysis

SPSS software (Version 11, SPSS, Inc., Chicargo, IL, USA) was used for statistical analysis. Data were expressed as mean ± SEM. Differences in measured values among the multiple groups were analyzed using analysis of variance with Bonferroni’s multiple comparison test. The chi square test was used to compare the frequency distribution between groups. Differences were considered significant when *p* < 0.05.

## SUPPLEMENTARY MATERIALS FIGURE



## Abbreviations

I/R: cerebral ischemia/reperfusion injury
4-HNE: 4-Hydroxynonenal
MDA: Malondialdehyde
TH: tyrosine hydroxylase
