# Supplementary material for: mir-193 targets ALDH2 and contributes to toxic aldehyde accumulation and tyrosine hydroxylase dysfunction in cerebral ischemia/reperfusion injury
Source: Oncotarget. 2017 Sep 21;8(59):99681–92. doi: 10.18632/oncotarget.21129 (PMC5725124; doi:10.18632/oncotarget.21129)
Supplement: Supplementary file 1 [file oncotarget-08-99681-s001.pdf]

mir-193 targets ALDH2 and contributes to toxic aldehyde accumulation and tyrosine hydroxylase dysfunction in cerebral ischemia/reperfusion injury

SUPPLEMENTARY MATERIALS

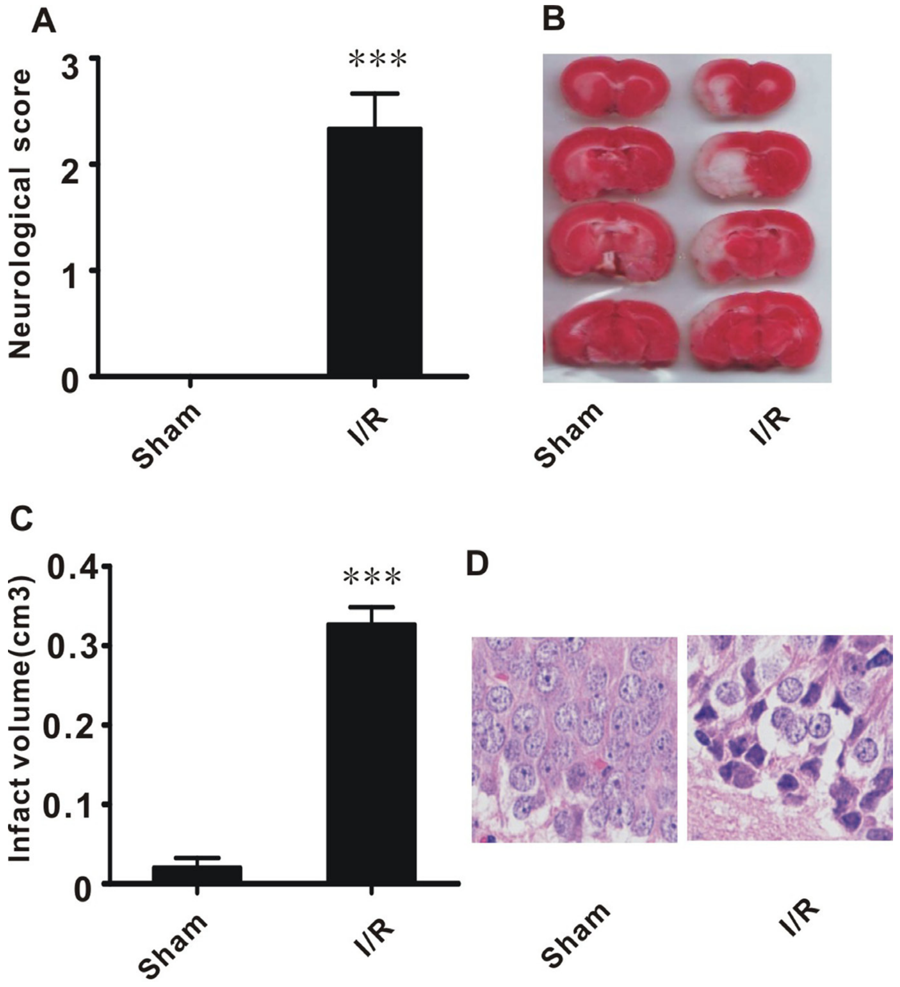

Supplementary Figure 1: Cerebral ischemia/reperfusion induced brain tissue injury. (**A**) Neurological deficit score in each group (n = 8); (**B**) representative image of triphenyltetrazolium chloride-stained brain tissue from each group (red area represents non-infarction, white area represents infarction); (**C**) infarct volume in each group (n = 8); (**D**) representative image of H&E stained brain tissue from each group. All values were expressed as means ±S.E.M. I/R: ischemia/reperfusion. $^{***}p < 0.001$ vs sham.
